# Anterior Vertebral Body Tethering Versus Posterior Spinal Fusion in Adolescent Idiopathic Scoliosis: A Systematic Review and Meta-Analysis of Comparative Outcomes

**DOI:** 10.3390/jcm14196707

**Published:** 2025-09-23

**Authors:** Mohamed Abdelaal, Maher Ghandour, Ümit Mert, Miguel Pishnamaz, Matthias Knobe, Frank Hildebrand, Rolf Sobottke, Koroush Kabir, Mohamad Agha Mahmoud

**Affiliations:** 1 Department of Orthopedics, Trauma and Reconstructive Surgery, University Hospital RWTH, 52074 Aachen, Germany; drabdelaalmohamed@gmail.com (M.A.); mghandourmd@gmail.com (M.G.); mpishnamaz@ukaachen.de (M.P.); fhildebrand@ukaachen.de (F.H.); 2 Department of Trauma and Orthopaedic Surgery, Helios University Hospital Wuppertal, University of Witten/Herdecke, 42283 Wuppertal, Germany; uemit.mert@helios-gesundheit.de (Ü.M.); koroush.kabir@helios-gesundheit.de (K.K.); 3 Department of Traumatology, St. Marien Hospital Ahaus, 48683 Ahaus, Germany; knobema@gmail.com; 4 Department of Spine, Neuro- and Orthopedic Surgery, Rhein-Maas Clinic, 52146 Würselen, Germany; sobottke@rheinmaasklinikum.de

**Keywords:** vertebral body tethering, spinal fusion, adolescent idiopathic scoliosis, scoliosis surgery, meta-analysis

## Abstract

**Background/Objectives:** To compare the radiographic, perioperative, and patient-reported outcomes between anterior vertebral body tethering (VBT) and posterior spinal fusion (PSF) in adolescents with idiopathic scoliosis. **Methods:** A systematic search of PubMed, Scopus, Web of Science, and Google Scholar was performed through May 2025. Studies directly comparing anterior VBT and PSF in skeletally immature patients with adolescent idiopathic scoliosis were included. Data were pooled using random-effects meta-analysis and expressed as mean differences (MDs) or odds ratios (ORs) with 95% confidence intervals (CIs). The NIH quality assessment tool was used to evaluate risk of bias. **Results:** Ten studies comprising 1168 patients (573 VBT, 595 PSF) were included. At 2 years, VBT showed a significantly greater main thoracic curve (MD = 5.03°; 95% CI: 1.87–8.20) and proximal thoracic curve (MD = 3.27°; 95% CI: 1.16–5.38), but no difference in lumbar or main curve Cobb angles. VBT was associated with significantly reduced thoracic kyphosis (MD = −2.68°), increased T1 tilt (MD = 1.50°), shorter operative time (MD = −99.23 min), less blood loss (MD = −405.44 mL), and shorter hospital stay (MD = −1.34 days). However, VBT had a significantly higher revision rate (OR = 5.54; 95% CI: 2.81–10.94). No significant differences were noted in SRS-22 domains, except for higher mental health scores in the VBT group (MD = 0.56; 95% CI: 0.07–1.06). **Conclusions:** Anterior VBT offers perioperative advantages and comparable radiographic correction to PSF in selected adolescents with idiopathic scoliosis, but at the cost of higher revision rates.

## 1. Introduction

Adolescent idiopathic scoliosis (AIS) is the most common spinal deformity in growing children, affecting approximately 2–3% of adolescents worldwide [1,2]. While most cases are mild and managed conservatively, progressive curves—especially those exceeding 45–50 degrees—often require surgical intervention to prevent long-term morbidity [3]. For decades, posterior spinal fusion (PSF) has remained the gold standard for operative management, offering reliable curve correction and halting progression [4]. However, PSF sacrifices spinal motion at the fused segments and has been associated with stiffness, adjacent segment degeneration, and activity restrictions in young, active patients [5].

In response to the limitations of fusion, anterior vertebral body tethering (VBT) has emerged as a novel, motion-preserving alternative for selected skeletally immature patients [6]. By applying growth modulation through tensioned anterior tethers, VBT aims to gradually correct the deformity while maintaining spinal mobility. Despite promising early results and increasing adoption, concerns persist regarding its long-term durability, rate of curve progression [7], and need for reoperations—particularly as many candidates for VBT remain several years from skeletal maturity.

VBT is typically considered for skeletally immature patients with AIS who have remaining growth potential (e.g., Sanders ≤ 5 or Risser 0–2), flexible main thoracic or thoracolumbar curves, and a Cobb magnitude generally in the ~35–65° range, particularly when bracing has failed or is not tolerated [8,9,10,11]. VBT aims to harness growth modulation to correct deformity while preserving segmental motion; however, it carries risks of over- or under-correction and a non-trivial likelihood of subsequent revision [12]. By contrast, PSF remains the standard for larger and/or more rigid curves and for patients nearing or at skeletal maturity; PSF provides reliable three-dimensional correction and durability but at the cost of segmental motion loss and potential adjacent-segment effects over time [8,9,10,11]. Framing our comparison within these typical indications underscores that VBT and PSF often address overlapping but not identical clinical scenarios, which is relevant to interpreting peri-operative outcomes, curve correction, and revision risk

Although several comparative studies have evaluated VBT versus PSF, their findings are inconsistent, limited by small sample sizes, variable follow-up durations, and heterogeneity in outcome reporting [8,9,10,11]. This systematic review and meta-analysis aims to address this critical gap by evaluating the current comparative evidence on VBT versus PSF in adolescents with idiopathic scoliosis, focusing on spinal correction, perioperative parameters, revision risk, and functional outcomes.

## 2. Materials and Methods

### 2.1. Protocol Registration and Reporting Standards

This systematic review and meta-analysis was conducted in accordance with the Preferred Reporting Items for Systematic Reviews and Meta-Analyses (PRISMA) 2020 guidelines [13]. The review protocol was developed prospectively and registered in the International Prospective Register of Systematic Reviews (PROSPERO) [Registration ID: CRD420250653261]. All stages of study identification, selection, data extraction, and quality assessment were performed in duplicate to ensure methodological rigor and reproducibility [14].

### 2.2. Literature Search Strategy

A comprehensive and systematic search was conducted across the following electronic databases: PubMed, Scopus, Web of Science, and Google Scholar, from inception to 11 May 2025. The search strategy combined MeSH terms and free-text keywords related to “adolescent idiopathic scoliosis”, “vertebral body tethering”, “posterior spinal fusion”, and “fusion surgery”. The complete search syntax is available in Supplementary Table S1. Reference lists of all included studies and relevant reviews were manually screened for additional eligible articles [15].

### 2.3. Study Selection

Eligible studies were those that directly compared anterior VBT and PSF in skeletally immature patients with adolescent idiopathic scoliosis. Inclusion criteria were (1) comparative cohort or case–control designs (prospective or retrospective); (2) reporting at least one relevant radiographic, clinical, or patient-reported outcome; and (3) a minimum of 10 patients per arm. Exclusion criteria were case reports, reviews, studies without a comparison group, studies not reporting extractable outcome data, and non-human or cadaveric studies. Studies that involved combined anterior and posterior tethering (double tethering) were excluded to maintain homogeneity, as our analysis was restricted to isolated anterior thoracic VBT compared with PSF. Two independent reviewers screened titles and abstracts using Microsoft Excel, followed by full-text review. Disagreements were resolved by consensus or adjudicated by a third reviewer.

### 2.4. Data Extraction and Outcome Definitions

A standardized data extraction form was used to collect study characteristics (author, year, country, study design, sample size), demographic data (age, sex, curve magnitude), surgical details (instrumentation levels, fusion vs. tethering), and outcome data. Outcomes were categorized into primary, secondary, and clinical/patient-reported endpoints a priori. Primary outcomes included main thoracic curve correction, proximal thoracic curve, lumbar curve, and main curve Cobb angle. Secondary outcomes encompassed lumbar Cobb angle, sagittal lordosis, thoracic kyphosis, and T1 tilt. Clinical endpoints included operative time, intraoperative blood loss, length of hospital stay, opioid use, revision surgery, and SRS-22 scores. When studies reported outcomes at multiple time points, data were extracted for each relevant follow-up period. If data were only available in graphical form, numeric values were extracted using WebPlotDigitizer (v4.6) [16]. Where necessary, corresponding authors were contacted to provide missing or clarifying data.

### 2.5. Risk of Bias Assessment

The methodological quality of included studies was assessed using the NIH Quality Assessment Tool for Observational Cohort and Cross-Sectional Studies, which evaluates internal validity across 14 domains, including selection, confounding, measurement, and attrition [17]. Each study was independently assessed by two reviewers and rated as good, fair, or poor quality. Disagreements were resolved through discussion.

### 2.6. Data Synthesis and Statistical Analysis

Where data were reported as median and interquartile range (IQR) or median and range, these were converted to mean and standard deviation (SD) using established statistical formulas recommended by the Cochrane Handbook [18,19]. Meta-analyses were conducted using STATA (v18.0, StataCorp LLC, College Station, TX, USA). Continuous outcomes were pooled as mean differences (MDs) with 95% confidence intervals (CIs), and binary outcomes as odds ratios (ORs) using the random-effects model, given the anticipated heterogeneity. Heterogeneity was quantified using the I^2^ statistic, with thresholds of 25%, 50%, and 75% indicating low, moderate, and high heterogeneity, respectively. A *p*-value < 0.10 in the Cochran Q test was considered statistically significant for heterogeneity. In instances where heterogeneity was negligible (I^2^ = 0% or *p*-value > 0.10) and the assumption of homogeneity was reasonable, a fixed-effects model was applied. Subgroup analyses were performed for key timepoints (e.g., 6 weeks, 3 months, 1 year, 2 years) when multiple studies reported the same outcome. Sensitivity analyses were conducted by excluding studies with outlying results or high risk of bias. Publication bias was not formally assessed due to the limited number of studies per outcome (<10) [20].

## 3. Results

### 3.1. Literature Search Results

**Figure 1:**
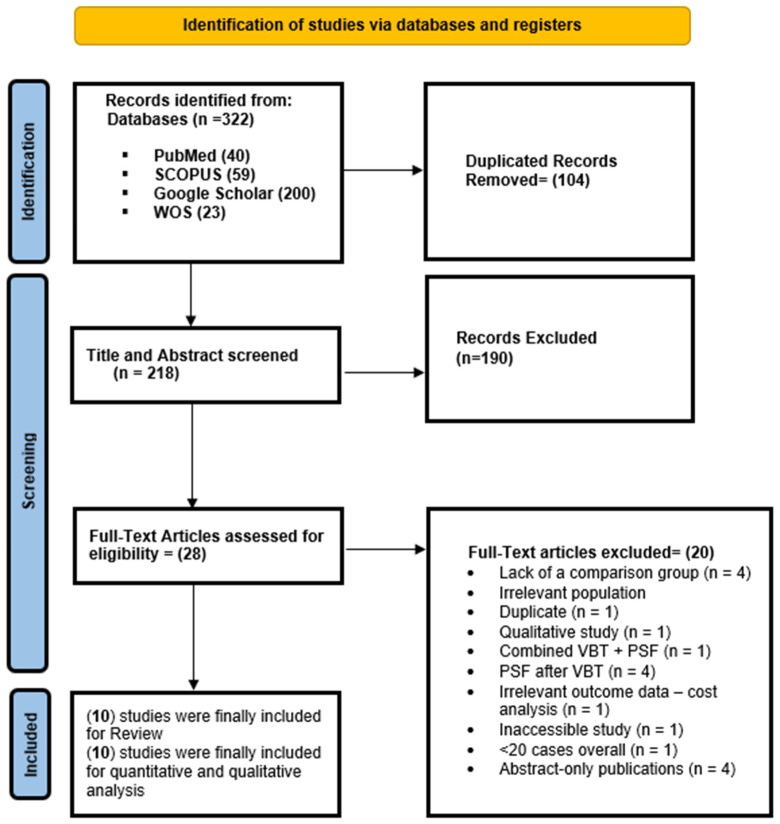
A PRISMA flow diagram showing the results of the database search.

A PRISMA flow diagram is shown in Figure 1. A total of 322 records were initially identified from four electronic databases: PubMed (n = 40), Scopus (n = 59), Google Scholar (n = 200), and Web of Science (n = 23). After removing 104 duplicate entries, 218 unique records remained for title and abstract screening. Of these, 190 records were excluded based on irrelevance to the study question. The remaining 28 full-text articles were assessed for eligibility. Twenty studies were excluded for the following reasons: lack of a comparison group (n = 4), irrelevant population (n = 4), duplicate (n = 1), qualitative study design (n = 1), combined VBT and PSF intervention (n = 1), PSF performed after VBT (n = 4), irrelevant outcome data such as cost analysis (n = 1), inaccessible full-text (n = 1), small sample size < 20 cases (n = 1), and abstract-only publications (n = 4). Ultimately, 10 studies met the eligibility criteria and were included in both qualitative and quantitative synthesis [8,9,10,11,21,22,23,24,25,26].

### 3.2. Baseline Characteristics of Included Studies

A summary of the baseline data of included studies can be found in Table 1. Most evidence came from the United States (8 studies) followed by Turkey (2 studies). Overall, 1168 patients with adolescent idiopathic scoliosis were examined, of whom 573 patients underwent anterior VBT and 595 underwent PSF. The mean patients’ age in the anterior VBT group was 13.91 years while that of the PSF group was 13.13 years. The pooled male-to-female ratio was 1.41:1 in the anterior VBT group and 1.39 in the PSF group; however, in one study [25], patients were mostly males (97.92%). The preoperative mean main thoracic curve was 55.9 in the anterior VBT group and 50.08 in the PSF group.

### 3.3. Methodological Quality of Included Studies

A summary of the methodological quality of included studies using the NIH tool for cohort studies is provided in Table 2. Overall, only three studies had good quality, while the remaining studies had fair quality. This was due to the lack of sample size justification, low sample sizes, and lack of confounding adjustment.

### 3.4. Primary Endpoints

#### 3.4.1. Main Thoracic Curve

**Figure 2:**
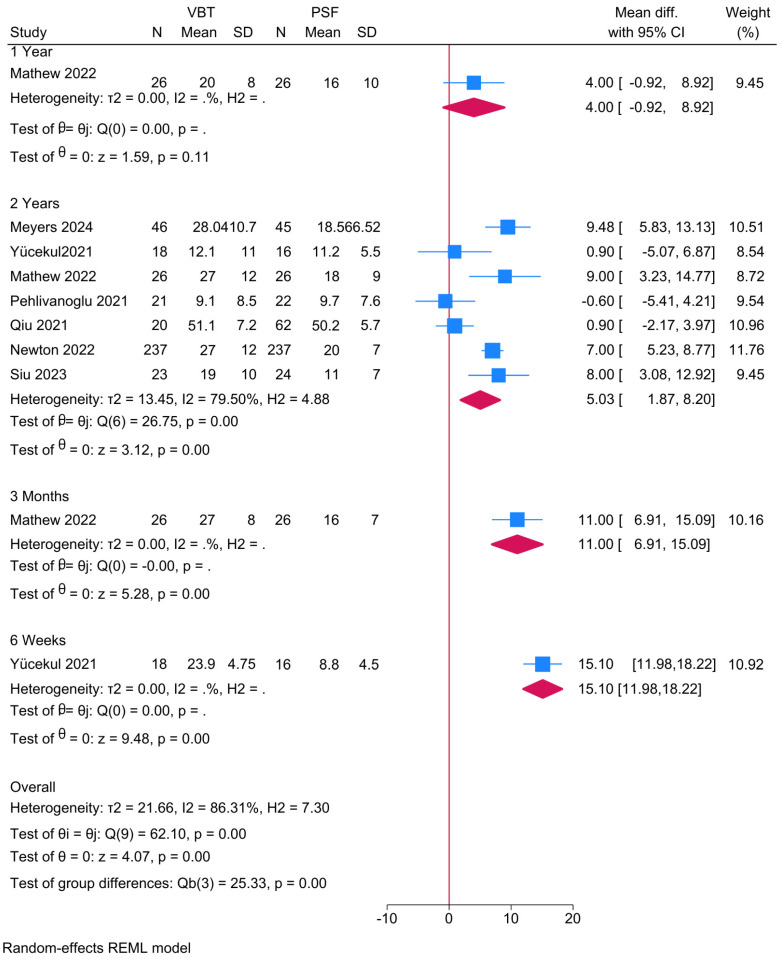
Forest plot showing the difference in main thoracic curve between PSF and VBT [9,10,11,22,23,25,26].

Seven studies were meta-analyzed and, given the different assessment timepoints, a subgroup was created showing a significantly greater main thoracic curve angle in the anterior VBT group compared to PSF at 2 years (MD = 5.03; 95% CI: 1.87–8.20; I^2^ = 79.50%, *p* < 0.001) (Figure 2). Although a similar observation was noted at 6 weeks and 3 months, these were deemed inconclusive given the small sample.

#### 3.4.2. Proximal Thoracic Curve

**Figure 3:**
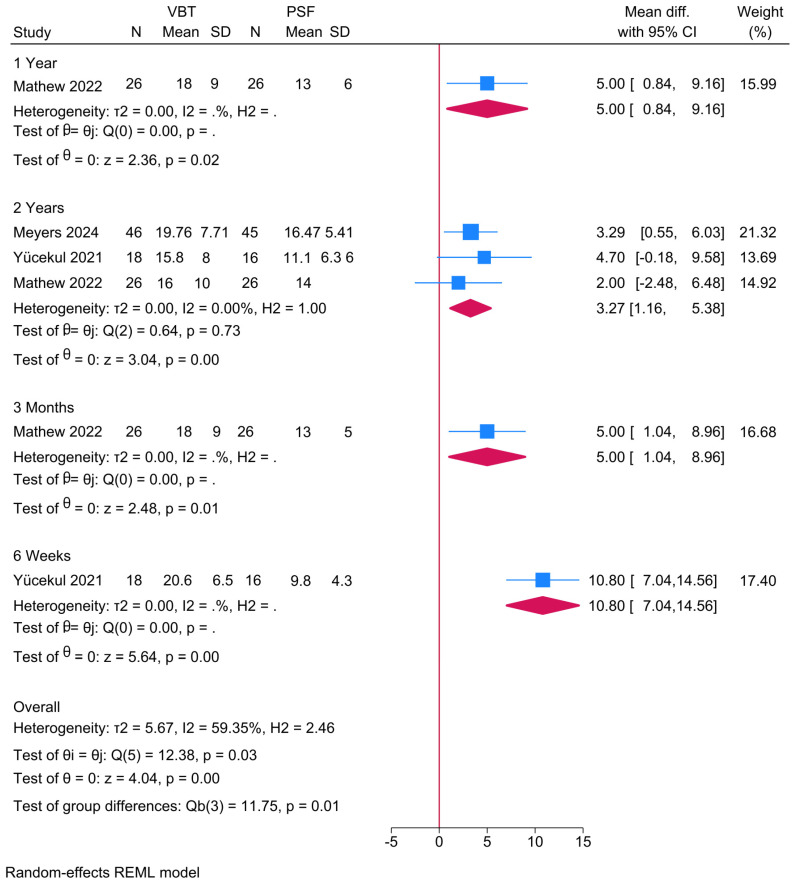
Forest plot showing the difference in proximal thoracic curve between PSF and VBT [9,22,26].

Three studies were meta-analyzed and, given the different assessment timepoints, a subgroup was created showing a significantly greater proximal thoracic curve angle in the anterior VBT group compared to PSF at 2 years (MD = 3.27; 95% CI: 1.16–5.38; I^2^ = 0%, *p* = 0.73) (Figure 3). Although similar observations were noted at 6 weeks, 3 months, and 1 year, these were deemed inconclusive given the small sample.

#### 3.4.3. Lumbar (Lumbo-Thoracic) Curve

Four studies were meta-analyzed and, given the different assessment timepoints, a subgroup was created showing no difference in the lumbo-thoracic curve angle between the anterior VBT group and PSF at 2 years (MD = 2.75; 95% CI: −3.28, 8.79; I^2^ = 86.94%, *p* < 0.001) (Figure S1). Although a greater angle was noted in the anterior VBT group at 6 weeks (MD = 4.40), this was deemed inconclusive given the small sample.

#### 3.4.4. Main Curve (Thoracic) Cobb

**Figure 4:**
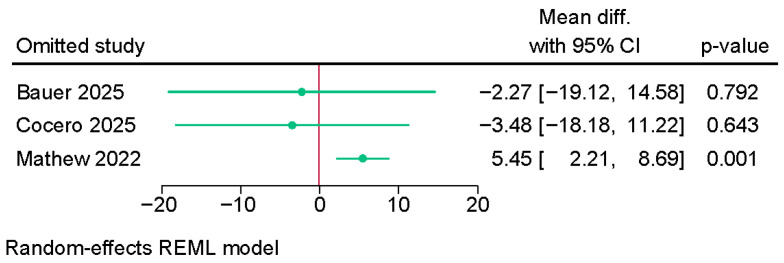
Sensitivity analysis of the difference in main curve (thoracic) Cobb angle between PSF and VBT [8,9,21].

Three studies were meta-analyzed, showing no difference in the main curve Cobb angle between the anterior VBT group and PSF (MD = −0.14; 95% CI: −10.66, 10.38; I^2^ = 92.28%, *p* < 0.001) (Figure S2). The sensitivity analysis, however, showed an increased Cobb angle in the anterior VBT group after excluding the study of Mathew et al. [9] (MD = 5.45; 95% CI: 2.21, 8.69) (Figure 4).

### 3.5. Secondary Endpoints

#### 3.5.1. Lumbar Cobb

**Figure 5:**
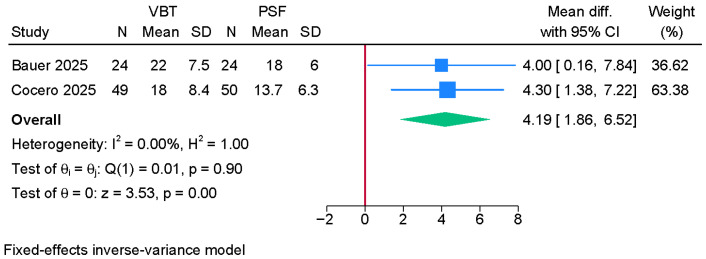
Forest plot showing the difference in lumbar Cobb angle between PSF and VBT [8,21].

Two studies were meta-analyzed, showing a significantly greater lumbar Cobb angle in the anterior VBT group over PSF (MD = 4.19; 95% CI: 1.86, 6.52; I^2^ = 0%, *p* = 0.90) (Figure 5).

#### 3.5.2. Sagittal Lordosis

Three studies were meta-analyzed, showing no difference in the lumbar Cobb angle between the anterior VBT group and PSF (MD = −5.42; 95% CI: −12.85, 2.02; I^2^ = 78.37%, *p* = 0.01) (Figure S3). The sensitivity analysis revealed no change in the reported estimate (Figure S4).

#### 3.5.3. Thoracic Kyphosis

**Figure 6:**
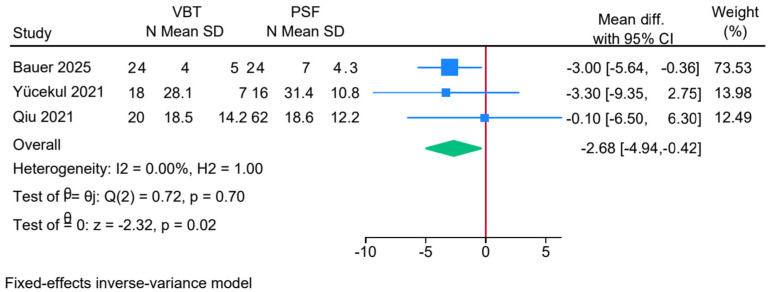
Forest plot showing the difference in thoracic kyphosis between PSF and VBT [8,11,26].

Three studies were meta-analyzed, showing a significantly reduced thoracic kyphosis angle in the anterior VBT group compared to the PSF group (MD = −2.68; 95% CI: −4.94, −0.42; I^2^ = 0%, *p* = 0.70) (Figure 6).

#### 3.5.4. T1 Tilt

**Figure 7:**
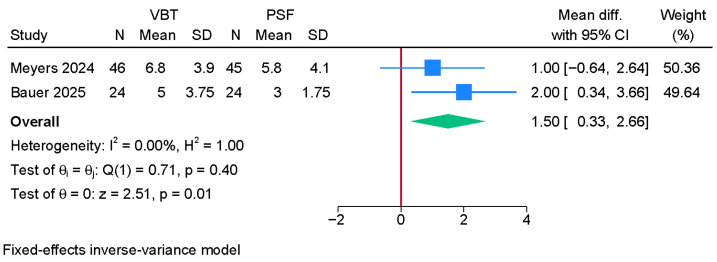
Forest plot showing the difference in T1 tilt between PSF and VBT [8,22].

Two studies were meta-analyzed, showing a significantly greater T1 degree tilt in the anterior VBT group over PSF (MD = 1.50; 95% CI: 0.33, 2.66; I^2^ = 0%, *p* = 0.40) (Figure 7).

### 3.6. Clinical and Patient-Reported Outcomes

#### 3.6.1. Operative Time (mins)

**Figure 8:**
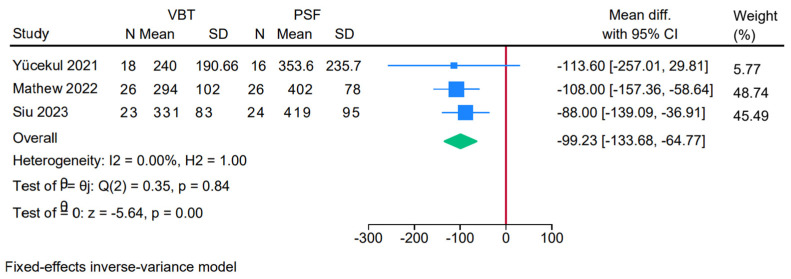
Forest plot showing the difference in operative time between PSF and VBT [9,25,26].

Three studies were meta-analyzed, showing a significantly shorter operative time in the anterior VBT group compared to PSF (MD = −99.23; 95% CI: −133.68, −64.77; I^2^ = 0%, *p* = 0.84) (Figure 8).

#### 3.6.2. Amount of Blood Loss (mL)

Three studies were meta-analyzed, showing a significantly lesser amount of blood loss in the anterior VBT group compared to PSF (MD = −405.44; 95% CI: −723.09, −87.80; I^2^ = 89.79%, *p* < 0.001) (Figure S5). The sensitivity analysis, however, showed no difference between both groups after excluding the study of Siu et al. [25] (Figure S6).

#### 3.6.3. Length of Hospital Stay

Three studies were meta-analyzed, showing a significantly shorter length of hospital stay in the anterior VBT group compared to PSF (MD = −1.34; 95% CI: −1.77, −0.91; I^2^ = 0%, *p* = 0.67) (Figure S7).

#### 3.6.4. Opiate Morphine Equivalent (OME)

Two studies were meta-analyzed, showing a significantly lower OME in the anterior VBT group compared to PSF (MD = −2.58; 95% CI: −3.40, −1.77; I^2^ = 27.99%, *p* = 0.24) (Figure S8).

#### 3.6.5. Revision Surgery Rate

**Figure 9:**
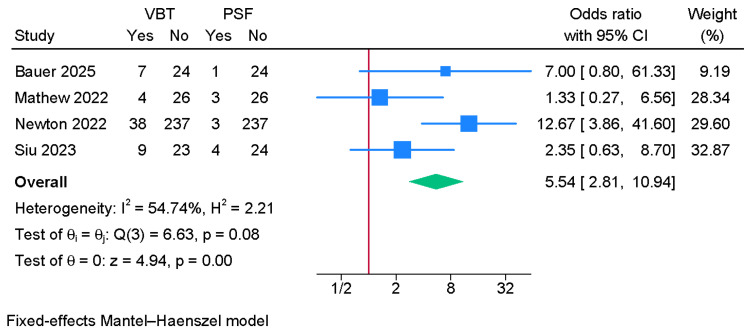
Forest plot showing the difference in revision surgery rate between PSF and VBT [8,9,10,25].

Four studies were meta-analyzed, showing a significantly greater risk of revision surgery in the anterior VBT group compared to PSF (OR = 5.54; 95% CI: 2.81, 10.94, I^2^ = 54.74%, *p* = 0.08) (Figure 9).

#### 3.6.6. Scoliosis Research Society-22 (SRS-22)

**Figure 10:**
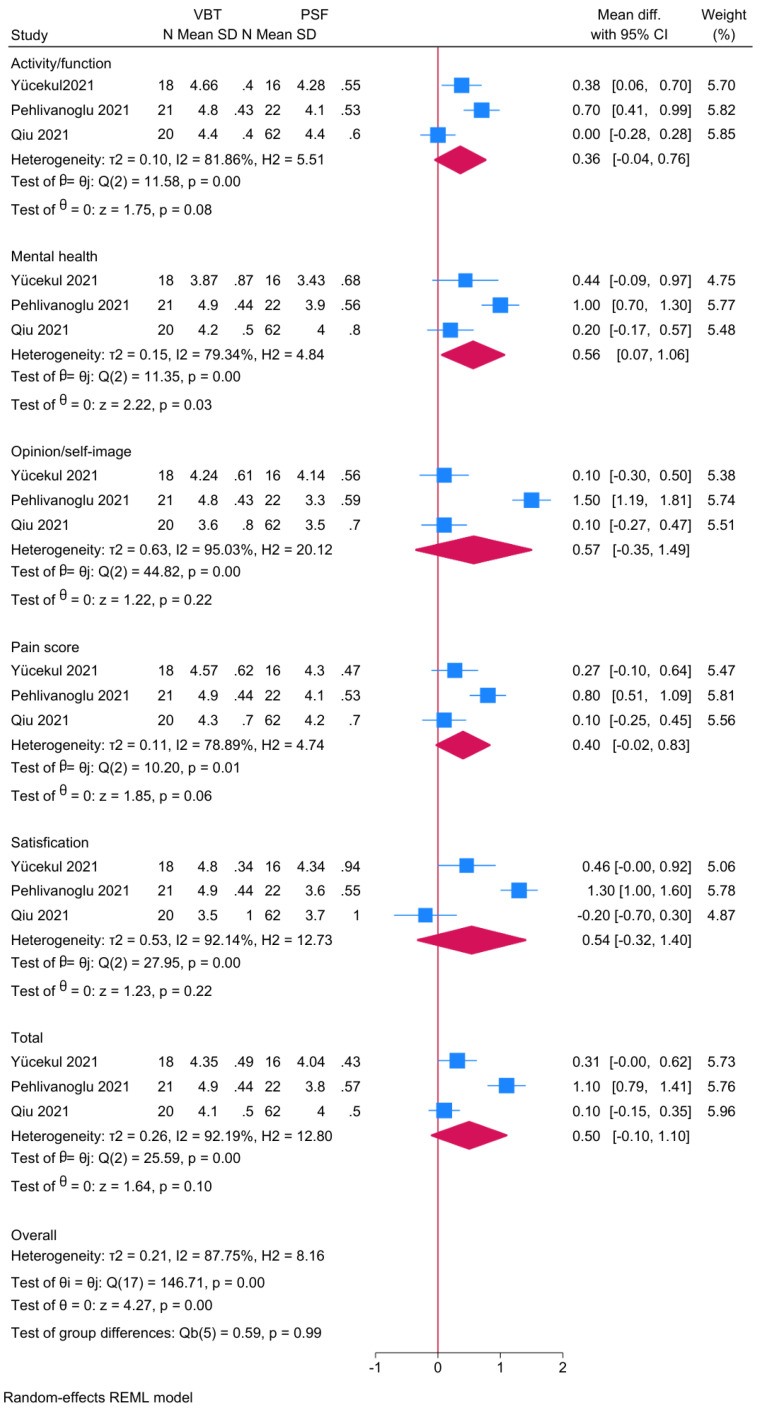
Forest plot showing the difference in scoliosis research society-22 (SRS-22) score between PSF and VBT [11,23,26].

Three studies were meta-analyzed (Figure 10). No significant differences between anterior VBT and PSF were noted in regards to the total SRS-22 score, satisfaction, pain score, opinion/self-image, or activity/function. However, the anterior VBT was associated with a significantly higher mental health score compared to PSF (MD = 0.56; 95% CI: 0.07, 1.06; I^2^ = 79.34%, *p* < 0.001). 

## 4. Discussion

This systematic review and meta-analysis offer the most comprehensive comparison to date between anterior VBT and PSF for the treatment of AIS. Our findings suggest that while VBT achieves comparable curve correction in certain spinal regions and yields advantages in operative morbidity and recovery metrics, it is also associated with higher revision rates and less consistent radiographic outcomes. These results underscore the nuanced trade-offs between these two surgical modalities, with important implications for patient-centered decision-making.

### 4.1. Curve Correction and Radiographic Outcomes

The main thoracic and proximal thoracic curves were significantly greater at two years in the VBT group compared to PSF, indicating less curve correction in the former. This aligns with prior observations suggesting that the corrective power of VBT may be inherently limited by its dependence on remaining spinal growth and gradual modulation rather than immediate mechanical correction. Our findings are consistent with prior scholars [27,28,29], who noted that while VBT preserves spinal mobility, it may be associated with less robust radiographic correction compared to fusion-based strategies.

Interestingly, the Cobb angle of the main curve did not significantly differ between groups in pooled analysis, though sensitivity analysis revealed a significant advantage for VBT when the study by Mathew et al. [9] was excluded. This suggests that study-level heterogeneity—possibly related to surgical technique, patient maturity, or curve characteristics—plays a crucial role in outcome interpretation [27]. Similar variability has been reported in recent prospective cohorts, including Oeiding et al. [30] and Pehlivanoglu et al. [23], emphasizing the need for standardization of surgical indications and protocols.

The lumbar curve and lumbar Cobb angle were significantly greater in the VBT group, which may reflect the intentional preservation of lumbar mobility and the avoidance of stiff instrumentation in this region [29]. However, the clinical significance of these residual curves remains uncertain and warrants longer-term evaluation.

### 4.2. Sagittal Parameters and Spinal Balance

Thoracic kyphosis was significantly reduced in the VBT group, raising concerns about iatrogenic hypokyphosis. This finding is consistent with previous literature highlighting kyphosis flattening as a potential drawback of anterior tethering techniques [31]. Lordosis and sagittal alignment did not differ significantly between groups, but the long-term consequences of these sagittal shifts—especially with respect to adjacent segment degeneration—remain unclear and should be a focus of future longitudinal studies.

Pelvic and coronal alignment metrics, such as T1 tilt and CSVL deviation, were inconsistently reported and varied substantially across studies, limiting definitive conclusions. Nevertheless, the significantly greater T1 tilt in the VBT group may reflect imbalanced correction dynamics and warrants careful radiographic planning during surgery [32].

Our pooled analysis showed that VBT was associated with reduced thoracic kyphosis compared with PSF at 2-year follow-up. From a biomechanical perspective, this is noteworthy because VBT is predicated on the principle of growth modulation. By applying compressive forces across the anterior vertebral column, the tether is expected to slow anterior growth relative to the posterior column, thereby correcting the coronal deformity while theoretically maintaining or even restoring kyphosis. However, in practice, hypokyphosis has frequently been reported after VBT. This paradox likely reflects the anterior location of the tether construct, which may restrict anterior column height and promote a flat-back tendency, particularly in the thoracic spine. Moreover, patient selection plays a role: skeletally immature patients with more flexible curves may be predisposed to over-correction in the sagittal plane as their growth potential is harnessed. By contrast, PSF—through posterior instrumentation and contouring of rods—tends to re-establish or even increase kyphosis, albeit at the cost of motion across fused segments. These principles help explain our observation that VBT preserves coronal mobility but risks sagittal hypokyphosis, whereas PSF provides more reliable sagittal alignment at the expense of motion.

An additional consideration is motion preservation, a frequently cited rationale for VBT. While tethering does avoid segmental fusion, the overall functional implications may be more nuanced. Pays et al. [33] reported that although segmental range of motion was better maintained after VBT compared with PSF, total trunk motion was not significantly different between VBT and selective thoracic fusion groups, and patient-reported outcomes were comparable at 2-year follow-up. These findings suggest that the theoretical motion-preserving advantage of VBT may not fully translate into measurable differences in global trunk mobility or short-term quality of life. Longer-term follow-up and more comprehensive functional assessments will be necessary to determine whether motion preservation yields sustained clinical benefits.

### 4.3. Operative Metrics and Clinical Recovery

A clear advantage of VBT lies in perioperative morbidity. We found significantly lower operative time, blood loss, and hospital stay in the VBT cohort, corroborating reports from Pehlivanoglu et al. [23] and Ahmad et al., which have consistently highlighted the less invasive nature of tethering approaches. Reduced opioid requirements postoperatively further support the favorable recovery profile associated with VBT. These differences are particularly relevant for patients and families prioritizing quicker return to function and shorter rehabilitation.

Despite these advantages, the revision surgery rate was significantly higher in the VBT group, with over a fivefold increase in odds compared to PSF. Reported causes include tether breakage, overcorrection, and loss of modulation, as well as technical failures [34]. This limitation reflects the intrinsic dependency of VBT on growth modulation, making it vulnerable to both under- and overcorrection, especially in patients with unpredictable skeletal maturity trajectories [35].

### 4.4. Patient-Reported Outcomes

While no significant differences were detected in most SRS-22 domains, mental health scores were significantly higher among VBT patients. This may reflect improved body image, preserved flexibility, and psychological benefits of undergoing a motion-sparing procedure, especially in younger adolescents [11]. However, these advantages should be interpreted with caution, as follow-up durations were limited and patient expectations may introduce bias.

### 4.5. Limitations and Future Directions

Our review is limited by the predominantly retrospective design of the included studies and the moderate-to-high heterogeneity observed across several outcomes. With few studies informing several outcomes, both I^2^ and Q-test *p*-values should be interpreted cautiously due to small-sample instability. In this context, meta-regression was not pursued (insufficient studies per covariate), and we did not present prediction intervals because τ^2^ is imprecisely estimated with very small k. While we employed robust sensitivity analyses and subgrouping by timepoint, variations in surgical technique, tethering systems, skeletal maturity, and outcome definitions remain major sources of bias. Furthermore, the limited duration of follow-up in most studies precludes definitive conclusions about long-term efficacy, durability, and complication rates—particularly given the risk of late tether failure or overcorrection. Emphasizing the longest common follow-up mitigates short-term transients, but longer, uniformly timed studies are needed to refine durability estimates.

Moreover, complication outcomes could not be meta-analyzed because only two studies reported them in sufficient detail, and inconsistent definitions with potential double-counting of multiple events per patient would have risked biased estimates.

Importantly, the surgical indications for VBT and PSF differ. VBT is generally reserved for skeletally immature patients with flexible curves and substantial growth remaining, whereas PSF remains the treatment of choice for patients with larger or more rigid curves, or for those approaching skeletal maturity. This distinction underscores that outcomes may partly reflect inherent differences in candidate selection in addition to surgical technique.

Future prospective studies with standardized outcome reporting and long-term follow-up are urgently needed. In particular, greater emphasis should be placed on identifying patient phenotypes most likely to benefit from VBT, refining surgical indications, and developing predictive models for growth modulation. Randomized trials may not be feasible due to strong treatment preferences, but large prospective registries and multicenter collaborations could generate high-quality evidence.

### 4.6. Clinical Implications

The choice between VBT and PSF must be individualized, balancing the benefits of motion preservation and quicker recovery against the risk of suboptimal correction and revision. VBT may be more appropriate for skeletally immature patients with flexible curves and strong preference for motion preservation. Conversely, PSF remains the gold standard for achieving durable, immediate correction, particularly in patients nearing skeletal maturity or with rigid deformities.

## 5. Conclusions

In this systematic review and meta-analysis, anterior VBT was associated with shorter operative time, reduced blood loss, quicker postoperative recovery, and comparable patient-reported outcomes compared to PSF in adolescents with idiopathic scoliosis. However, VBT demonstrated less consistent radiographic correction and a significantly higher revision surgery rate. While VBT may offer advantages in selected patients, PSF remains the more reliable option for durable curve correction. Long-term comparative data are needed to better define the role of VBT in the surgical management of adolescent idiopathic scoliosis.

## Figures and Tables

**Table 1:** Baseline characteristics of included studies comparing anterior VBT to PSF.

Study ID	Study Design	YOI	Country	Sample Size		Age; Mean (SD)		Female (%)	
				VBT	PSF	VBT	PSF	VBT	PSF
Meyers 2024 [22]	Retrospective cohort	2024	USA	46	45	12.8 (2.1)	13.5 (2.2)	89.1%	91.1%
Bauer 2025 [8]	Prospective cohort	2025	USA	24	24	13.5	14	-	-
Varona-Cocero 2025 [21]	Retrospective cohort	2025	USA	49	50	13.6 (1.4)	13.2 (1.9)	84%	97.2%
Yücekul 2021 [26]	Retrospective cohort	2014–2019	Turkey	18	16	15.7(2)	13.4 (1.75)	94.4%	81.3%
Mathew 2022 [9]	Prospective cohort	2022	USA	26	26	13.2 (0.23)	13.4 (0.2)	88.5%	88.5%
Pehlivanoglu 2021 [23]	Retrospective cohort	2021	Turkey	21	22	11.1 (1.5)	10.9 (1.5)	71.4%	72.7%
Pulido 2025 [24]	Retrospective cohort	2024	USA	109	89	13.3 (2.3)	14.8 (2.5)	82.6%	85.4%
Qiu 2021 [11]	Prospective cohort	2017–2020	USA	20	62	11.8 (1.9)	11.7 (0.9)	80%	87%
Newton 2022 [10]	Retrospective cohort	-	USA	237	237	12.1 (1.6)	13.4 (1.4)	83.97%	83.97%
Siu 2023 [25]	Retrospective cohort	2014–2019	USA	23	24	12 (1)	13 (1)	87%	92%

YOI: year of investigation; VBT: vertebral body tethering; PSF: posterior spinal fusion; SD: standard deviation.

**Table 2:** A summary of the methodological quality of included studies using the National Institute of Health tool for cohort studies.

ID	Q1	Q2	Q3	Q4	Q5	Q6	Q7	Q8	Q9	Q10	Q11	Q12	Q13	Q14	Total Score	Overall Rating
Meyers 2024 [22]	1	2	2	1	0	1	2	2	2	1	2	1	2	0	19	Fair
Bauer 2025 [8]	2	2	2	1	0	2	2	2	2	1	2	1	2	0	21	Good
Varona-Cocero 2025 [21]	1	1	2	1	0	1	2	2	2	1	2	1	1	0	17	Fair
Yücekul 2021 [26]	2	2	2	1	0	1	2	2	2	2	2	1	1	0	20	Fair
Mathew 2022 [9]	2	2	2	1	0	2	2	2	2	2	2	1	2	0	22	Good
Pehlivanoglu 2021 [23]	1	1	2	1	0	1	2	2	2	1	2	1	0	0	16	Fair
Pulido 2025 [24]	2	2	2	1	0	1	2	2	2	1	2	1	0	0	18	Fair
Qiu 2021 [11]	1	1	2	1	0	2	2	2	2	1	2	1	0	0	17	Fair
Newton 2022 [10]	2	2	2	1	0	2	2	2	2	2	2	1	2	0	22	Good
Siu 2023 [25]	1	1	2	1	0	2	2	2	2	1	2	1	0	0	17	Fair

1. Was the research question or objective in this paper clearly stated? 2. Was the study population clearly specified and defined? 3. Was the participation rate of eligible persons at least 50%? 4. Were all the subjects selected or recruited from the same or similar populations (including the same time period)? Were inclusion and exclusion criteria for being in the study prespecified and applied uniformly to all participants? 5. Was a sample size justification, power description, or variance and effect estimate provided? 6. For the analyses in this paper, was the exposure(s) of interest measured prior to the outcome(s) being measured? 7. Was the timeframe sufficient so that one could reasonably expect to see an association between exposure and outcome if it existed? 8. For exposures that can vary in amount or level, did the study examine different levels of the exposure as related to the outcome (e.g., categories of exposure, or exposure measured as continuous variable)? 9. Were the exposure measures (independent variables) clearly defined, valid, reliable, and implemented consistently across all study participants? 10. Was the exposure(s) assessed more than once over time? 11. Were the outcome measures (dependent variables) clearly defined, valid, reliable, and implemented consistently across all study participants? 12. Were the outcome assessors blinded to the exposure status of participants? 13. Was loss to follow-up after baseline 20% or less? 14. Were key potential confounding variables measured and adjusted statistically for their impact on the relationship between exposure(s) and outcome(s)? Good quality = score > 20; Fair quality = score 15–20; Poor quality = score < 15.

## Data Availability

Data are within the manuscript and its Supplementary Materials.

## Supplementary Materials

The following supporting information can be downloaded at https://www.mdpi.com/article/10.3390/jcm14196707/s1, Table S1. The detailed search query employed in the database search; Figure S1. Forest plot showing the difference in lumbar (lumbo-thoracic) curve between PSF and VBT; Figure S2. Forest plot showing the difference in main curve (thoracic) Cobb angle between PSF and VBT; Figure S3. Forest plot showing the difference in sagittal lordosis between PSF and VBT; Figure S4. Sensitivity analysis of the difference in sagittal lordosis between PSF and VBT; Figure S5. Forest plot showing the difference in amount of blood loss between PSF and VBT; Figure S6. Sensitivity analysis of the difference in amount of blood loss between PSF and VBT; Figure S7. Forest plot showing the difference in length of hospital stay between PSF and VBT; Figure S8. Forest plot showing the difference in opiate morphine equivalent (OME) between PSF and VBT.

## Abbreviations

The following abbreviations are used in this manuscript:

AIS	Adolescent Idiopathic Scoliosis
PSF	Posterior Spinal Fusion
VBT	Vertebral Body Tethering
MD	Mean Difference
OR	Odds Ratio
CI	Confidence Interval
SRS-22	Scoliosis Research Society-22 Questionnaire
SD	Standard Deviation
IQR	Interquartile Range
NIH	National Institutes of Health
CSVL	Central Sacral Vertical Line
OME	Opiate Morphine Equivalent
PRISMA	Preferred Reporting Items for Systematic Reviews and Meta-Analyses
PROSPERO	International Prospective Register of Systematic Reviews
RCT	Randomized Controlled Trial
