# Supplementary material for: Anterior Vertebral Body Tethering Versus Posterior Spinal Fusion in Adolescent Idiopathic Scoliosis: A Systematic Review and Meta-Analysis of Comparative Outcomes
Source: J Clin Med. 2025 Sep 23;14(19):6707. doi: 10.3390/jcm14196707 (PMC12525045; doi:10.3390/jcm14196707)
Supplement: Supplementary file 1 [file jcm-14-06707-s001.zip › Table S1.pdf]

Table S1. The detailed search query employed in the database search

No.	Search syntax	Results
**PubMed**		
**#1**	"spinal fusion"[tiab] OR "spinal fusions"[tiab] OR spondylodesis[tiab] OR spondylodeses[tiab] OR spondylosyndesis[tiab]	11712
**#2**	Posterior[tiab]	358684
**#3**	Adolescen*[tiab]	419160
**#4**	"idiopathic scoliosis"[tiab]	9899
**#5**	"***Vertebral body tethering***"[tiab]	206
**#6**	#1 AND #2 AND #3 AND #4 AND #5	40
**Scopus**		
**#1**	TITLE-ABS-KEY ("spinal fusion") OR TITLE-ABS-KEY ("spinal fusions") OR TITLE-ABS-KEY (spondylodesis) OR TITLE-ABS-KEY (spondylodeses) OR TITLE-ABS-KEY (spondylosyndesis)	39220
**#2**	TITLE-ABS-KEY (Posterior)	516603
**#3**	TITLE-ABS-KEY (Adolescen*)	279906 1
**#4**	TITLE-ABS-KEY ("idiopathic scoliosis")	13112
**#5**	TITLE-ABS-KEY ("***Vertebral body tethering***")	228
**#6**	#1 AND #2 AND #3 AND #4 AND #5	59
**Web of Science**		
**#1**	AB="spinal fusion" OR AB="spinal fusions" OR AB=spondylodesis OR AB=spondylodeses OR AB=spondylosyndesis	8041
**#2**	AB=Posterior	348634
**#3**	AB=Adolescen*	380743
**#4**	AB="idiopathic scoliosis"	7470
**#5**	AB="***Vertebral body tethering***"	174
**#6**	#1 AND #2 AND #3 AND #4 AND #5	23
**Google Scholar**		
**With all of the words**	Posterior Spinal fusion vertebral body tethering	-
**With the exact phrase**	Idiopathic scoliosis	-
**With at least one of the words**	Adolescent adolescence	-
**Total**	-	200
