# Supplementary figures and images for: Anterior Vertebral Body Tethering Versus Posterior Spinal Fusion in Adolescent Idiopathic Scoliosis: A Systematic Review and Meta-Analysis of Comparative Outcomes
Source: J Clin Med. 2025 Sep 23;14(19):6707. doi: 10.3390/jcm14196707 (PMC12525045; doi:10.3390/jcm14196707)

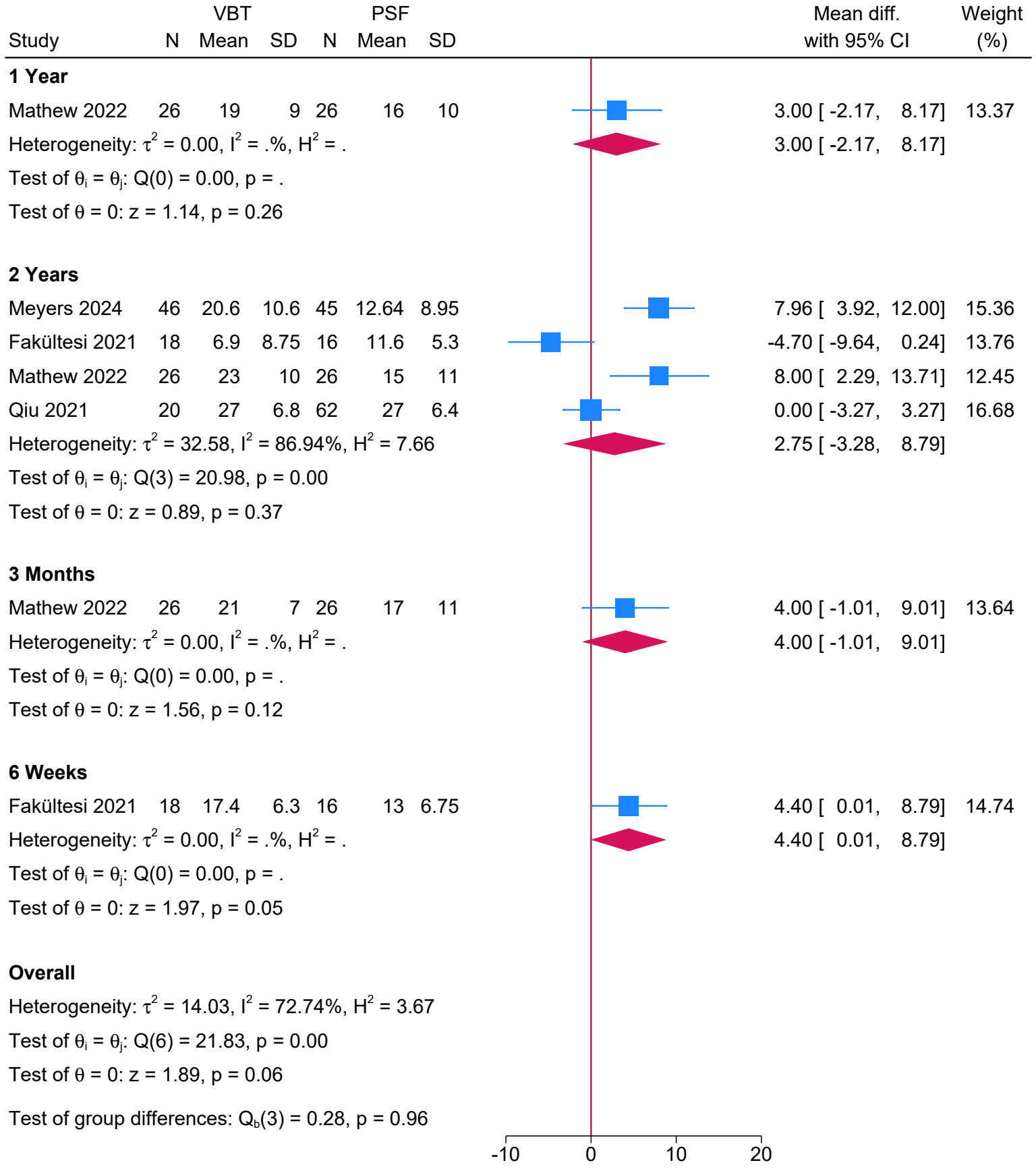

Supplement: Supplementary file 1 [file jcm-14-06707-s001.zip › Figure S1.pdf]

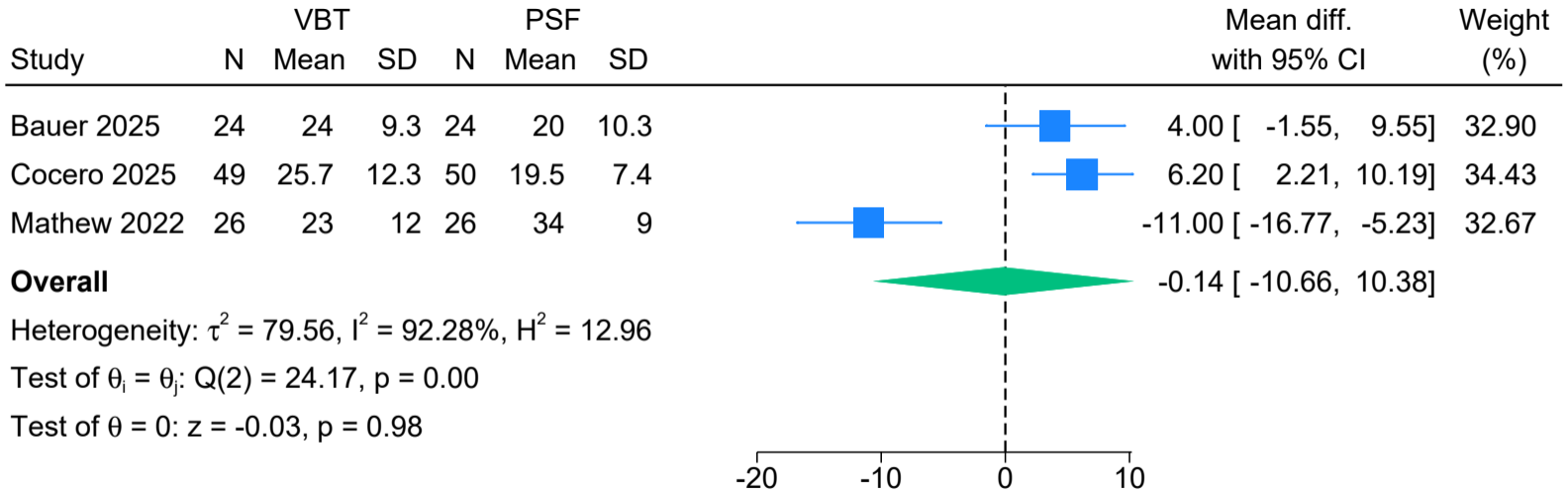

Random-effects REML model

Supplement: Supplementary file 1 [file jcm-14-06707-s001.zip › Figure S2.pdf]

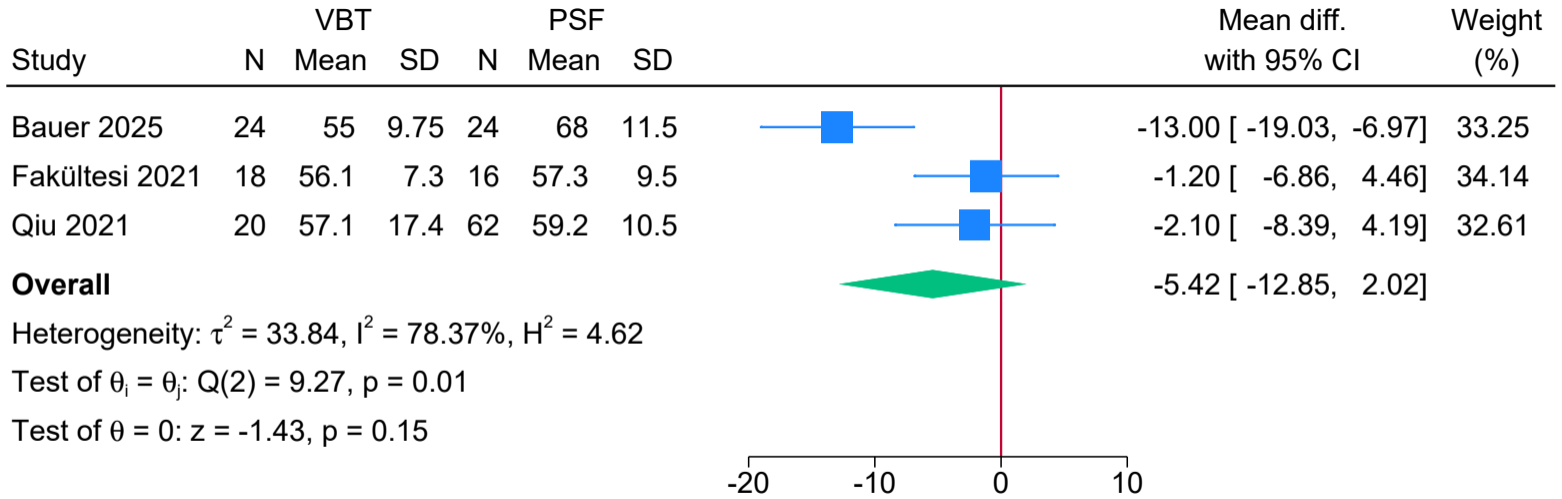

Random-effects REML model

Supplement: Supplementary file 1 [file jcm-14-06707-s001.zip › Figure S3.pdf]

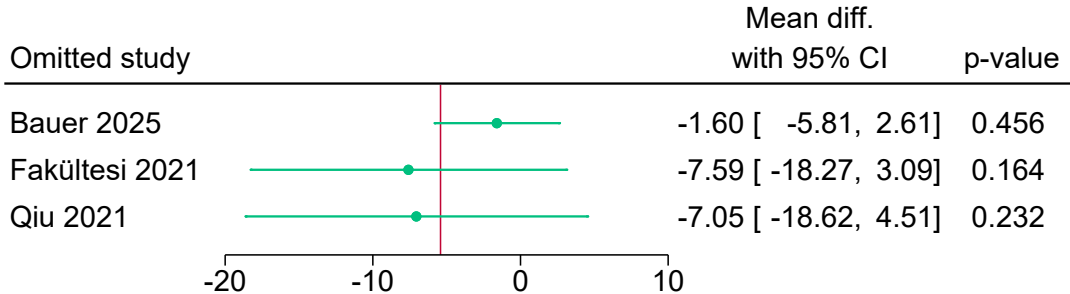

Random-effects REML model

Supplement: Supplementary file 1 [file jcm-14-06707-s001.zip › Figure S4.pdf]

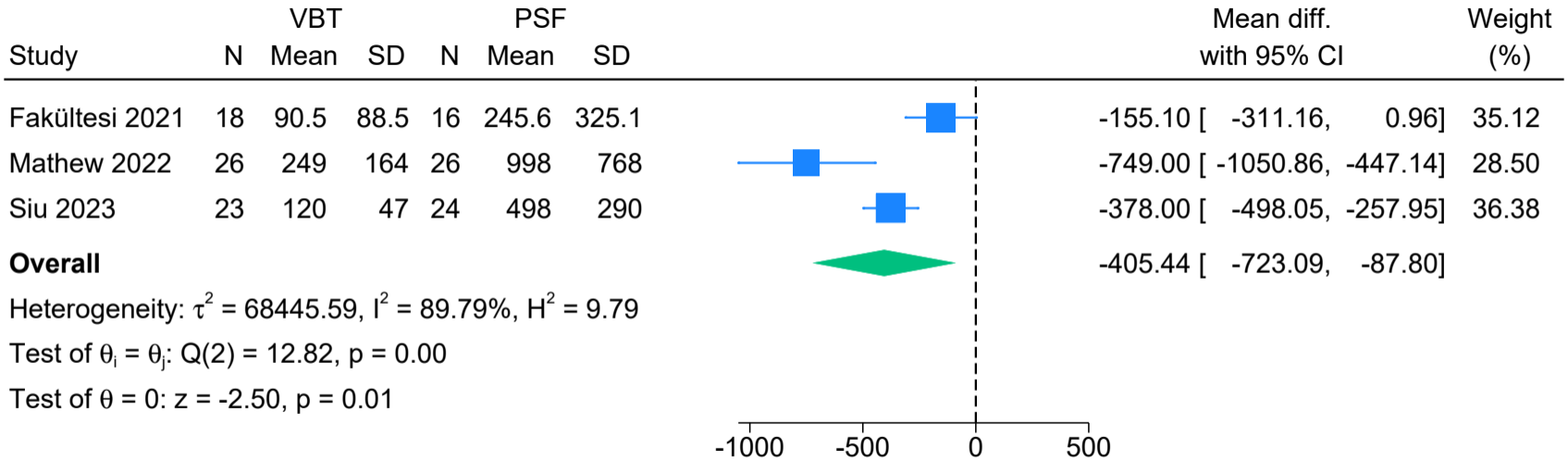

Random-effects REML model

Supplement: Supplementary file 1 [file jcm-14-06707-s001.zip › Figure S5.pdf]

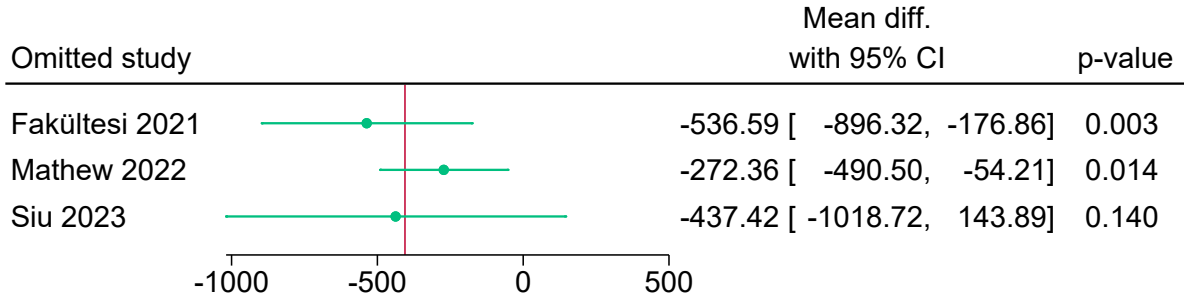

Random-effects REML model

Supplement: Supplementary file 1 [file jcm-14-06707-s001.zip › Figure S6.pdf]

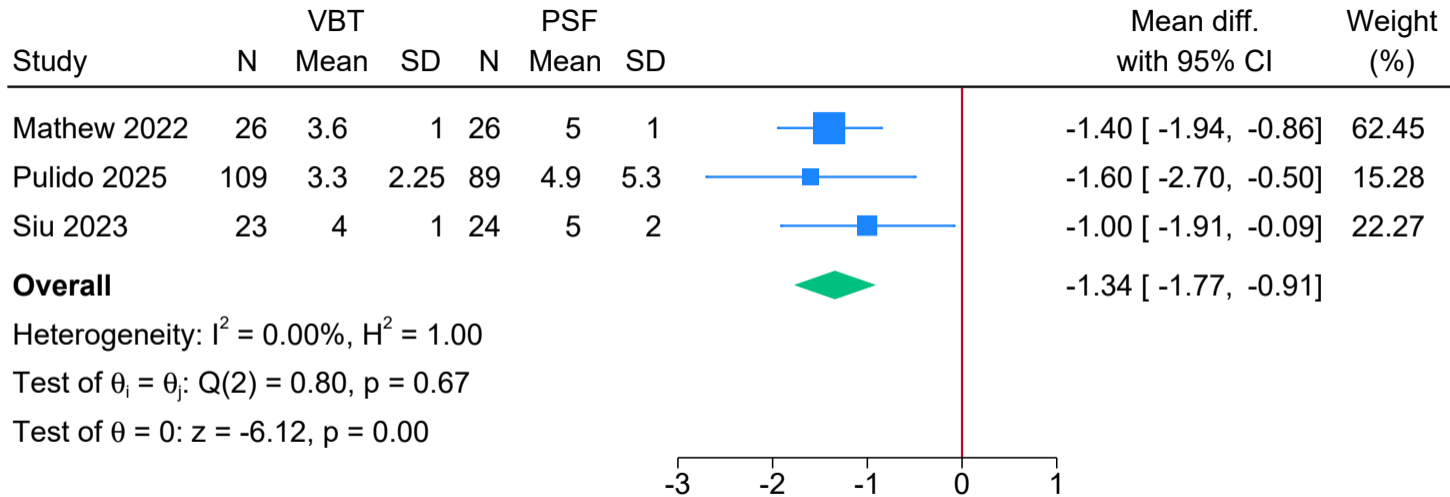

Fixed-effects inverse-variance model

Supplement: Supplementary file 1 [file jcm-14-06707-s001.zip › Figure S7.pdf]

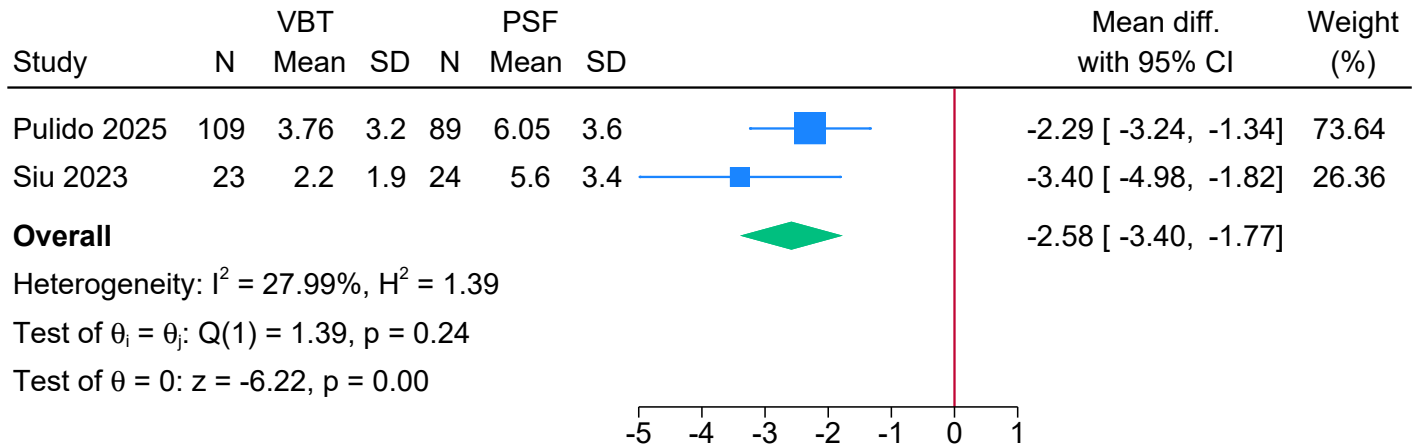

Fixed-effects inverse-variance model

Supplement: Supplementary file 1 [file jcm-14-06707-s001.zip › Figure S8.pdf]
